# The effect of four types of iron supplements on enamel microhardness of primary teeth: an in-vitro study

**DOI:** 10.1186/s12903-026-09414-z

**Published:** 2026-08-01

**Authors:** Parisa Vahedi, Somayeh Khoramian Tusi, Manijeh Mohammadian, Mahmood Sheikh Fathollahi, Zahra Mosayebi

**Affiliations:** 1 Department of Pediatric Dentistry, Alborz University of Medical Sciences, Karaj, Iran; 2 Department of Dental Biomaterials, Iran University of Medical Sciences, Tehran, Iran; 3 National Center for Health Insurance Research, Tehran, Iran; 4 Alborz University of Medical Sciences, Karaj, Iran

**Keywords:** Iron supplements, Enamel, Deciduous teeth, Pediatric Dentistry

## Abstract

**Background:**

Iron supplementation has been suggested as a potential factor influencing enamel properties; however, its direct effect on enamel microhardness remains unclear. The aim of this study was to evaluate the effect of four types of supplements on the microhardness of deciduous tooth enamel.

**Materials and methods:**

Fifty five healthy primary anterior teeth were randomly divided into five groups (*n* = 11): one control group and four experimental groups. The pH of four supplements (Liposofer, Feramax, Amivital Ferrous Sulphate and SiderAl) was recorded. Baseline enamel microhardness was assessed using a Vickers hardness tester (50 g load for 10 s). Samples of the control group were placed in 250 ml of distilled water and the samples of each experimental group were placed in a solution containing 250 ml of distilled water and a volume of iron supplements (equivalent to 100 mg of elemental iron) for 5 min in a Shaker Incubator. After rinsing, the microhardness of the enamel was measured again after the treatment. The mean microhardness of the deciduous teeth enamel was compared using One-way ANOVA across the studied groups.

**Results:**

One-way ANOVA demonstrated no statistically significant differences in mean enamel microhardness changes among the study groups (*p* > 0.05). The mean ± SD microhardness changes in the groups were as follows: control group 68.82 ± 68.13, Amivital Ferrous Sulphate group 32.06 ± 34.61, SiderAl group 84.45 ± 97.20, Liposofer group 30.35 ± 20.48, and Feramax group 58.58 ± 70.27.

**Conclusion:**

Within the limitations of this in-vitro study, short-term exposure to different iron supplements did not result in statistically significant differences in enamel microhardness of primary teeth. Although numerical differences were observed among the tested supplements, no statistically significant differences were detected.

## Introduction

The World Health Organization (WHO) reports that nearly two billion individuals, approximately 25% of the global population, experience anemia, with around half of them have Iron deficiency anemia (IDA) [1]. To prevent iron deficiency during early childhood, oral iron supplementation is commonly advised. A variety of oral iron supplements on the market are prescribed to those with IDA [2]. These supplements are often prescribed in the form of drops or syrups for ease of administration in infants and young children [3]. For infants and young children, the typical dose of elemental iron is 2 to 6 milligrams per kilogram, administered 1 to 3 times a day for a duration of 3 to 6 months [4].

Various reasons can cause this issue, such as the child’s refusal or dislike, the child’s stomach upset, and concerns about tooth decay [5, 6]. Some Persian parents mistakenly believe that iron supplements cause tooth decay [5]. Ferrous sulfate is considered the gold standard in oral iron supplements, as it is one of the cheapest and widely used formulations [7].

Iron supplementation may affect dental caries by reducing enamel demineralization and cariogenic bacterial activity, although the effects vary with the type and concentration of iron used [8, 9].

Deciduous teeth are more susceptible to acid-induced demineralization than permanent teeth due to their thinner enamel, lower mineral content and different structural properties [10, 11]. Enamel microhardness is a key mechanical parameter reflecting the resistance of enamel to wear and acid erosion and is commonly used to evaluate early demineralization process [12, 13].

The progression of oral iron supplementation has led to the development of newer iron formulations that aim to improve tolerability and safety [14]. These formulations include liposomal iron, sucrosomial iron, and polysaccharide–iron complexes, each of which differs in chemical structure, release mechanism, and interaction with biological tissues [15, 16].

Liquid iron supplements often contain acidic components to enhance stability and palatability, which may lower their pH and theoretically increase the risk of enamel demineralization when in direct contact with tooth surfaces [17–19]. Dental erosion is the chemical dissolution of the dental hard tissues by acids without the involvement of microorganisms. Hydrogen ions (H+) from acidic solutions can replace the calcium ions (Ca2+) of the enamel [20].

In fact, encapsulating iron in a micronized form in liposomes is considered a new approach to improve iron tolerance and absorption [21]. Sucrosomial iron (SI) symbolizes an innovative oral iron carrier in which ferric pyrophosphate is protected and transported across the intestinal mucosa by a phospholipid bilayer membrane [22]. Furthermore, polysaccharide iron complexes are prescribed as iron supplements for IDA [23].

Although several studies have investigated the effects of conventional ferrous sulfate preparations on dental tissues, limited evidence is available regarding the influence of newer iron formulations, including liposomal, sucrosomial, and polysaccharide-iron supplements, on the microhardness of primary enamel. Furthermore, direct comparisons among these formulations under standardized experimental conditions are lacking. Therefore, the present in-vitro study aimed to compare the effects of four commonly prescribed iron supplements (Amivital Ferrous Sulphate, Feramax, Liposofer, and SiderAl) on the enamel microhardness of primary teeth.

The null hypothesis of this study was that there would be no significant difference in enamel microhardness changes among primary teeth exposed to different types of iron supplements following short-term exposure.

## Materials and methods

The study has been approved by the Ethics Committee of Alborz University of Medical Sciences with the ethics approval code (IR.ABZUMS.REC.1403.205). Fifty-five extracted deciduous anterior teeth were selected [24]. Sample size was determined based on the study by Pasdar et al. [24], which evaluated enamel microhardness changes following exposure to iron-containing supplements. Considering an alpha error of 0.05, a study power of 80%, and the expected difference in microhardness values reported in the previous study, the minimum required sample size was estimated to be 10 specimens per group. To compensate for possible specimen loss during preparation and testing, 11 teeth were included in each group, resulting in a total sample size of 55 teeth. The extracted teeth were obtained from the Department of Pediatric Dentistry, Alborz University of medical sciences. All extractions were performed for therapeutic reasons (such as orthodontic indications or natural exfoliation) and not for the purpose of this study. Only teeth free from caries, cracks, fractures, restorations, and hypoplasia were included. The teeth were stored in 0.9% saline at room temperature [25]. The storage solution was refreshed weekly to prevent dehydration and microbial growth.

First, the crowns of the teeth were cut from the CEJ (cemento-enamel junction) and the pulp chamber contents were removed, and then the teeth were mounted in acrylic resin, which only the buccal surface of the teeth was exposed. The entire labial surface of the tooth was coated with nail polish, except for a square window in the middle third of the crown measuring 4 × 4 mm that was uncoated.

At this stage, the labial surface was polished with silicon carbide paper with grits of 600, 800, 1000, 1200, and 2000, respectively [24, 25] under water irrigation to standardize enamel surfaces.

In a Vickers hardness tester (ZwickRoell, ZHVµ, Ulm, Germany), a load of 50 g was applied to the teeth for 10 s [26]. To measure the initial microhardness of each sample, Three indentations were made per specimen and the mean value was recorded.

The specimens were randomly allocated to the five study groups using a computer-generated randomization sequence (*n* = 11). The operator who performed the final microhardness measurements was blinded to group allocation to minimize measurement bias. The samples of the control group were placed in a solution containing 250 ml of distilled water, and the samples of each experimental group were placed in a solution containing 250 ml of distilled water and a volume of iron supplements equivalent to 100 mg of elemental iron; 4 ml of Amivital Ferrous Sulphate (Amin Pharmaceutical Company, Iran), 4.6 g of Feramax iron powder (Behestan Health Company, Iran), 14.3 ml of Liposofer (Bonyan Salamat Kasra Company, Iran) and 14.3 ml of SiderAl (Junia Pharma, Italy) were placed in a Shaker Incubator (Heidolph, Unimax 1010, Germany) for 5 min at 37 °C [27, 28]. The reason for using 100 mg of iron in this study is that it matches the standard dose for treating iron deficiency anemia in children and shows the staining potential of iron solutions [27]. The contents of each supplement are described in Table 1.

**Table 1:** Types of consumed iron supplements

Iron supplement (company/country)	Type of Iron supplement	Ingredients
Liposofer (BSK, Iran)	Liposomal	30 ml of iron drops, each milliliter containing 7 mg of iron
Feramax (BioSyent Pharma Inc, Iran)	polysaccharide-iron	41.5 g of iron powder (60 scoops), each scoop containing 15 mg of iron
SiderAl (Junia Pharma, Italy)	sucrosomial	30 ml of iron drops combined with a 1.9-gram sachet, each milliliter containing 7 mg of iron
Amivital Ferrous Sulphate (Amin Pharma, Iran )	Ferrous Sulphate	15 ml of iron drops, each milliliter containing 125 mg of ferrous sulfate heptahydrate equivalent to 25 mg of iron and 3 mg of sodium saccharin

The pH of final immersion solutions (250 mL distilled water + iron supplement equivalent to 100 mg elemental iron) was measured once and recorded by using a digital pH meter (Adwa, AD1020, Warranty, Romania) at room temperature.

After 5 min, they were removed from the solution and washed with distilled water, and the secondary microhardness of all samples was measured again using a Vickers hardness tester [25]. The exposure time of 5 min was selected based on previous studies, which demonstrated that the greatest reduction in tooth hardness occurred after approximately 4 min of contact with acidic solutions [16].

Results are reported as mean ± standard deviation (SD). One-way analysis of variance (ANOVA) was used to compare the mean microhardness (initial microhardness, secondary microhardness, microhardness changes) of the deciduous tooth enamel across studied groups. The normality of the microhardness distribution of deciduous teeth enamel was assessed using the Shapiro-Wilk test, as well as calculating skewness and kurtosis indices. The homogeneity of the variance of the groups was also assessed using the Levene test. The collected data were statistically analyzed using SPSS version 27.0 (Statistical Package for the Social Sciences, IBM Inc., Armonk, NY, USA) software for Windows. Statistically significance level was set at 0.05.

## Results

The results of the laboratory analysis of the microhardness of the supplements are given in Table 2 and are discussed below.

**Table 2:** Vickers microhardness of primary tooth enamel after exposure to each experimental group

Supplements	Number	Baseline VHN (Mean ± SD)	Final VHN (Mean ± SD)	Mean ± SD (Decrease)	*P*-value (Initial)	*P*-value (Changes)	pH
Control	11	325.55 ± 37.34	256.73 ± 80.37	68.82 ± 68.13	0.614	0.107	-
Amivital Ferrous Sulphate	11	323.76 ± 43.70	291.70 ± 34.37	32.06 ± 34.61	0.114	0.261	2.24
SiderAl	11	340.30 ± 58.15	255.85 ± 90.37	84.45 ± 97.20	0.160	0.309	3.61
Liposofer	11	318.33 ± 29.74	283.03 ± 36.43	35.30 ± 20.48	0.683	0.085	3.43
Feramax	11	334.36 ± 37.32	275.79 ± 81.66	58.58 ± 70.27	0.232	0.187	7.67
*P*-value	-	0.755	0.674	0.275	0.755	0.275	-

The pH values of the test solutions exhibited considerable variation, ranging from 2.24 in Amivital Ferrous Sulphate solution to 7.67 in Feramax solution. Meanwhile, the pH levels of the other groups, specifically SiderAl and Liposofer, were recorded as 3.61 and 3.43, respectively.

**Fig. 1:**
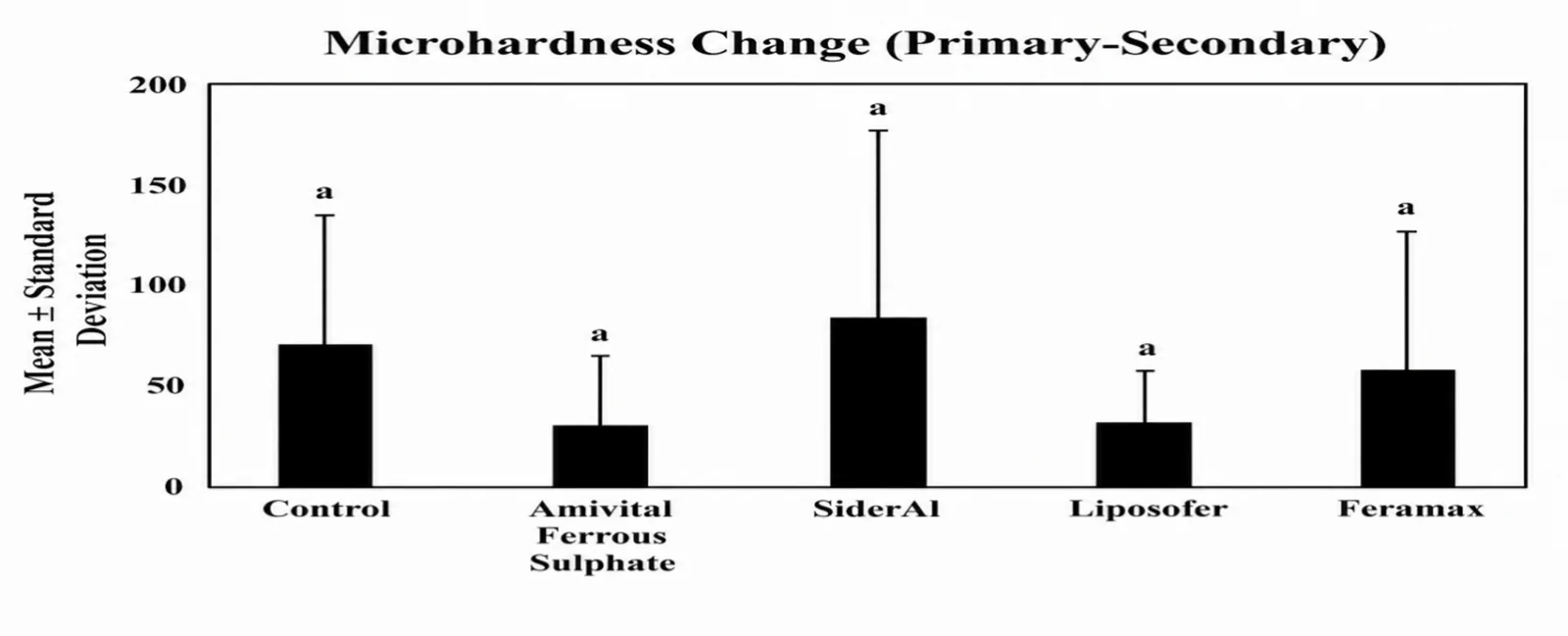
The average changes in microhardness (VHN) of primary tooth enamel by study groups. Groups sharing the same superscript letter (a) are not significantly different (*P* > 0.05)

The greatest numerical (though non-significant) reduction in microhardness was observed in the SiderAl group (84.45 ± 97.20) and the Amivital Ferrous Sulphate group had the lowest mean changes (32.06 ± 34.61) (Fig 1). Although differences were observed between the groups, these differences were not statistically significant (*p* > 0.05) (Table 2).

It can also be concluded from the results of the Shapiro-Wilk test that the changes in microhardness of the enamel of the deciduous teeth in each of the studied groups have a normal distribution (*p* > 0.05). The Levene statistical test also showed that the variance of the changes in microhardness of the enamel of the deciduous teeth in the studied groups is homogeneous (*p* > 0.05). Thus, the necessary assumptions for performing one-way ANOVA to compare the mean numerical changes in microhardness (primary-secondary) of deciduous tooth enamel according to the studied groups are established. The results of one-way analysis of variance showed that the mean changes in microhardness (VHN) of deciduous tooth enamel in the studied groups did not differ statistically significantly from each other (*p* > 0.05).

## Discussion

Iron deficiency anemia (IDA) is one of the most varieties of anemia globally [29]. The daily consumption of oral iron supplements is a widespread intervention for both treating and preventing IDA [30]. However, some parents refuse to use iron drops for their children due to the belief that such supplements may lead to dental cavities, despite their important role in child growth and development [28]. One way to prevent direct contact between iron drops and tooth surfaces is to use encapsulation technology, which makes controlled release of the contents inside the capsule [31, 32].

This in-vitro study evaluated the erosive potential of four different iron supplements on the microhardness of primary tooth enamel. To the best of our knowledge, this is one of the first studies to directly compare conventional ferrous sulfate with newer iron formulations, including liposomal, sucrosomial, and polysaccharide iron, using a standardized protocol and equivalent elemental iron concentration. The results of the present study showed that microhardness decreased in all four experimental groups after 5 min; The 5-minute exposure time was selected based on previous studies [16] indicating that the greatest reduction in tooth hardness occurs after approximately 4 min of contact with acidic solutions. In the present study, although a reduction in microhardness was observed among the groups, this decrease was not statistically significant either between the experimental groups or in comparison to the control group. This suggests that iron supplementation does not have a significant impact on the microhardness of the enamel surface in deciduous teeth. The lack of significant difference in microhardness may be due to the short exposure time, the dilution of supplements in distilled water. Moreover, the observed differences in microhardness reduction can be primarily attributed to the distinct chemical compositions and iron release mechanisms of the two supplements, despite the lower (more acidic) pH measured for Amivital Ferrous Sulphate.

Amivital, based on ferrous sulphate (Fe²⁺), releases free ferrous ions that have a well-documented protective effect on enamel. These ions react with dissolved phosphates to form a thin layer of ferric phosphate on the enamel surface, acting as an acid-resistant barrier that inhibits further demineralization and reduces microhardness loss, even in acidic environments [33].

In contrast, SiderAl utilizes sucrosomial iron (encapsulated ferric pyrophosphate in a phospholipid-sucrose matrix), which limits direct release of free iron ions. While this encapsulation reduces side effects like staining, it diminishes the protective phosphate precipitation effect. Additionally, SiderAl formulations often include citric acid for stability and taste, which increases titratable acidity and enhances erosive potential through prolonged low pH and chelation of calcium from hydroxyapatite [34].

Thus, the dominant protective action of freely released ferrous ions in Amivital overrides its lower initial pH, resulting in the least microhardness reduction, while the limited iron release and higher titratable acidity in SiderAl lead to the greatest average reduction—consistent with the non-significant overall differences observed in the study. However, these results may not fully reflect real-world oral conditions, as the study was conducted in vitro and did not account for the natural protective factors present in the mouth. The present study did not employ pH-cycling, which is considered a gold-standard laboratory method for simulating oral conditions. The decision to use a single 5-minute exposure was based on the aim of examining immediate effects under controlled conditions. Nevertheless, lack of pH-cycling may limit the ability to generalize findings to repeated or prolonged use of iron supplements in children.

The results obtained in the present study conform with the study by Eskandarian et al. [35], which explored the influence of three iron drops on the surface roughness of deciduous teeth in a simulated cariogenic environment. Their study discovered that the microhardness of enamel after contact with iron drops, at depths of 200 and 400 μm from the tooth surface, did not significantly change compared to a control group; however, the changes in microhardness in the group exposed to the cariogenic environment were significantly different from the control group; in other words, iron supplements do not impact the demineralization of the tooth structure. The similar results are due to the exposure of the samples to a non-cariogenic environment.

In addition, the study by Maden et al. [36], which investigated the effects of iron supplementation on the deciduous teeth of children aged 5 to 12 years, noted no significant differences in dental caries experience (DMFT) or iron levels in the enamel between those receiving iron supplements and those who did not; however, direct comparisons with the present study should be interpreted cautiously because dental caries and enamel microhardness represent different outcomes.

The findings of Eshghi and colleagues are consistent with this study. In their research, Eshghi et al. [37] examined the effect of iron supplementation on the advancement of dental caries in 12 rats. The results revealed that the occurrence of caries in the group receiving iron supplementation was lower compared to the groups fed diets lacking iron supplementation. These findings support the conclusion that iron supplementation does not exacerbate enamel demineralization. It is important to emphasize that enamel microhardness is an indirect indicator of mineral integrity and cannot be used alone to predict dental caries development. Dental caries is a multifactorial disease influenced by microbial, dietary, and host factors.

Concurrently, the study by Sahebnazar et al. [25], which analyzed the effect of sucrosomal iron and iron drops mixed with natural fruit juice on the microhardness of deciduous tooth enamel, reported that iron drops caused a significant decrease in enamel microhardness. Among the tested options, Irofant iron drops resulted in the greatest reduction in enamel microhardness. In contrast, samples exposed to SiderAl iron drops showed the least alterations. This discrepancy in results is likely due to differences in the amount of iron supplements in contact with the specimens. Moreover, the pH of the Irofant iron supplement was 1.98, which was lower than the pH of the supplements in the present study and the critical pH for enamel solubility.

Mukundan et al. [10] aimed to compare the effects of three pediatric syrups including iron drops, multivitamins, and urine syrup on the microhardness, roughness, and coloration of deciduous tooth enamel, reported all three liquid medications significantly altered the microhardness, roughness, and color of the tooth enamel compared to the control group over three weeks. Urine syrup had the strongest effect on microhardness and the iron drop had the least influence in comparison to the control group. The reason why the results of the present study are not consistent with the results mentioned above can be related to the difference in the contact time of the samples with the solutions and the different structure of the iron drops (using iron ascorbate). In iron ascorbate, iron will have higher absorption by binding to ascorbic acid, which was not present in the iron supplements of the present study. However, in the present study, the iron supplements used were one of each of the liposomal, sucrosomial, and polysaccharide iron supplements. In liposomal iron, the outer phospholipid layer protects the iron from being compromised by digestive enzymes and free radicals. Therefore, liposomes prevent oxidation and degradation of encapsulated iron [38]. In sucrosomial iron (SI), iron pyrophosphate is delivered via a phospholipid bilayer membrane [22]. Moreover, the samples in the mentioned study were exposed to the supplements for a longer duration compared to our research.

In the study of Hekmatfar et al. [39], which researched the correlation between the pH of five iron drops and their iron ion absorption in deciduous anterior teeth, it was noted that a low pH of iron drops may lead to tooth erosion, but iron can lessen enamel demineralization. One of the reasons for the difference in the results of this study and the present study is the difference in the experimental iron drops. FerroKids is an iron gluconate supplement. The other iron drops in the study were also ferrous sulfate. While in the present study, four different types of iron supplements were investigated. Ferrous sulfate may adhere to the enamel and can diffuse beyond the enamel, resulting in dark stains due to iron oxidation [40]. In contrast, the iron supplements tested here used an encapsulation method that prevents direct contact between the iron and tooth enamel.

One of the strengths of the present study was the uniformity of iron concentration across all groups (100 mg/250 mL), which allowed for ease of fair comparisons. Also, pH was measured as an important factor affecting enamel microhardness in this study. One of the limitations of this study was that the experiments were conducted in a laboratory setting, which did not fully simulate the oral environment. Saliva and its buffering capacity could significantly influence the final outcomes. Furthermore, the oral cavity is a favorable environment for the growth of microorganisms, which accumulate on dental surfaces to form dental plaque (oral biofilm). Among these, cariogenic bacteria ferment carbohydrates to produce acid, which can cause demineralization of tooth surfaces [41]. In this study, similar to previous studies [16, 25, 27], each tooth was exposed to iron supplements for 5 min. However, it is unclear whether exposure to iron drops for 5 min can adequately simulate the clinical setting. Despite these limitations, the results suggest that iron supplements, especially in encapsulated forms, are unlikely to adversely affect enamel microhardness in deciduous teeth. Dental practitioners can reassure parents regarding the safe use of iron drops, although long-term clinical studies are needed to confirm these findings.

## Conclusion

This in vitro study demonstrated that, despite differences in pH among the tested iron supplements, no significant changes in the microhardness of deciduous tooth enamel were observed. Findings of the present study suggest that short-term exposure to these supplements may not markedly affect enamel hardness under controlled laboratory conditions.

## Data Availability

The datasets used and/or analysed during this current study are available from the corresponding author on reasonable request.

## Abbreviations

CEJ: Cemento-enamel junction
DMFT: Decayed, missing, filled teeth
g: Gram
IDA: Iron deficiency anemia
ml: Milliliter
pH: Potential of hydrogen
SD: Standard Devitation
SI: Sucrosomial iron
VHN: Vickers hardness number
WHO: World Health Organization
